# Gastric Duplication Cyst: Two Case Reports and Review of the Literature

**DOI:** 10.1155/2013/605059

**Published:** 2013-02-19

**Authors:** Jai P. Singh, Heena Rajdeo, Kalyani Bhuta, John A. Savino

**Affiliations:** Department of Surgery, Westchester Medical Center, New York Medical College, Valhalla, NY 10595, USA

## Abstract

*Background*. Duplication of the alimentary tract is a rare congenital anomaly. Gastric duplication cysts (GDCs) represent 4% of all alimentary tract duplications, and approximately 67% manifest within the first year of life. Duplication cysts in adults are generally encountered as incidental findings at endoscopy or laparotomy. Herein, we report two rare cases of symptomatic GDC presenting in adults. *Case 1*. A 27-year-old male presented with a five-month history of back pain. Exam revealed mild epigastric tenderness with a vague palpable mass in left upper abdomen. CT scan showed 8 × 7.4 × 6 cm homogenous, nonseptated cystic mass posterosuperior to pancreatic tail. On laparotomy, a cystic mass measuring 11 × 8 cm was found, which was densely adherent to posterior wall of stomach suggestive of GDC. *Case 2*. A 28-year-old woman presented with epigastric pain associated with vomiting for 2 months. Exam revealed mild epigastric tenderness. CT scan showed four cystic lesions in the medial wall of distal stomach measuring approximately one cm each suggestive of duplication cysts. Exploratory laparotomy with antrectomy and truncal vagotomy with Billroth II reconstruction were performed. Pathology in both patients was diagnostic of GDC. *Conclusion*. GDC is a rare anomaly, and its presentation in adults is even rarer.

## 1. Introduction

Duplication of the alimentary tract is a relatively rare congenital anomaly. It can affect any part of the gastrointestinal tract with ileum being the most common site [1, 2]. These malformations are believed to be congenital, formed before the differentiation of epithelial lining, and therefore named for the organ with which they are associated [3]. Duplication cysts of the stomach represent four per cent of all alimentary tract duplications. Approximately 67 per cent of gastric duplication cysts (GDCs) are identified within the first year of life [4]. Duplication cysts in adults are generally asymptomatic and encountered as incidental findings at endoscopy or laparotomy [4]. Herein, we report two rare cases of symptomatic duplication cysts of stomach presenting in adults.

## 2. Case Reports

### 2.1. Case  1

A 27-year-old male presented with a five-month history of progressively increasing back pain associated with mild epigastric discomfort and loss of appetite. Review of systems revealed weight loss of approximately 25 pounds with occasional nausea. He denied vomiting and alteration of bowel habits. Family history was significant for the fact that his mother had been previously treated for benign cystic neoplasm of pancreas. Past medical history was not significant. Physical examination revealed mild epigastric tenderness with a vague palpable mass in the epigastric and left subcostal regions measuring approximately 7 × 5 cm.

**Figure 1:**
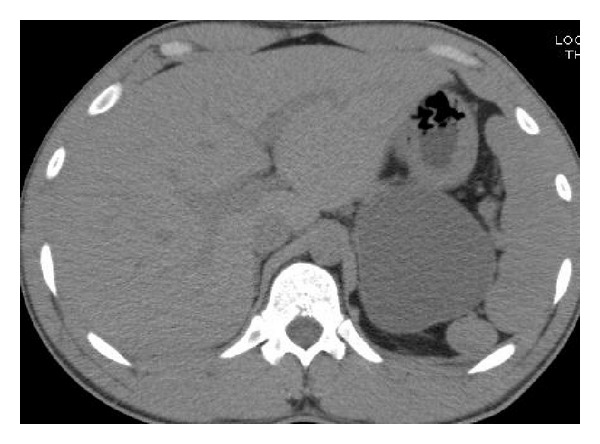
CT scan showing 8 × 7.4 × 6 cm homogenous, nonseptated cystic mass posterosuperior to pancreatic tail.

MRI and CT scans of the abdomen demonstrated 8 × 7.4 × 6 cm homogenous, nonseptated cystic mass posterosuperior to pancreatic tail (Figure 1). Left adrenal gland was not clearly identified. Pancreatic and biliary ducts were not dilated, and there was no evidence of any other mass or lymphadenopathy.

 Since it was not clear whether the mass was arising from adrenal or pancreas, a complete adrenal workup was done including 24-hour urinary cortisol, urinary VMA, metanephrine level, serum aldosterone, and renin, which did not reveal any evidence of functional adrenal tumor. 

 On exploratory laparotomy pancreas and left adrenal appeared normal; however there was a soft cystic mass measuring approximately 11 × 8 cm, which was densely adherent to posterior wall of stomach close to the greater curvature. Excision of cystic mass along with resection of adjoining stomach was performed for a presumed gastric duplication cyst.

**Figure 2:**
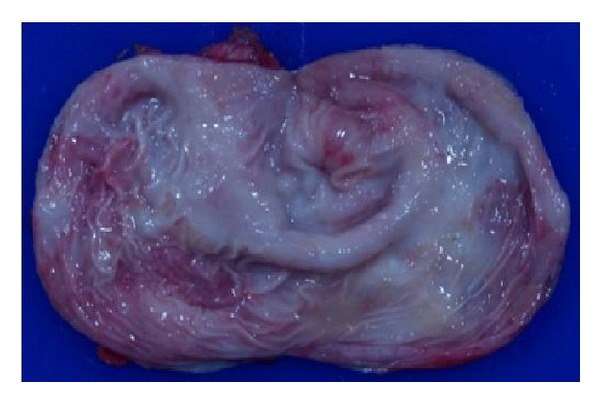
Inner surface of specimen was smooth and white-pink in color with a 1.5 × 1.2 cm rough area.

 Surgical specimen measured 10 × 7 cm. Cut surface of specimen revealed a light yellowish gelatinous material. The inner surface was smooth and white-pink in color with 1.5 × 1.2 cm rough area (Figure 2). There was no communication between cyst and resected gastric segment. 

 Patient's postoperative course was uneventful. He was discharged on postoperative day 4 and has been asymptomatic since then.

**Figure 3:**
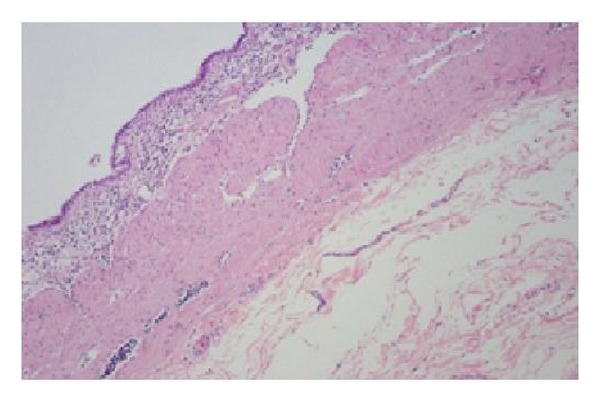
Photomicrograph: cyst wall was composed of mucosa, submucosa, and muscularis propria. The mucosa was predominantly a gastric body type with patchy intervening areas of simple columnar epithelium containing apical mucus and cilia seen in embryonic intestinal epithelium.

 On microscopy, cyst wall was composed of mucosa, submucosa, and muscularis propria with myenteric plexus. The mucosa was predominantly gastric body type consisting of parietal, chief, and mucus cells with patchy intervening areas of simple columnar epithelium containing apical mucus and cilia seen in embryonic intestinal epithelium (Figure 3).

### 2.2. Case  2

A 28-year-old woman was transferred from community hospital for evaluation of recurrent, nonradiating epigastric pain associated with nausea and occasional nonbilious vomiting for two months. She denied any change in bowel habits and weight loss. Her medical history was significant for lumbar herniated disc and recurrent shoulder dislocation. Physical exam was unremarkable except for mild epigastric tenderness. 

**Figure 4:**
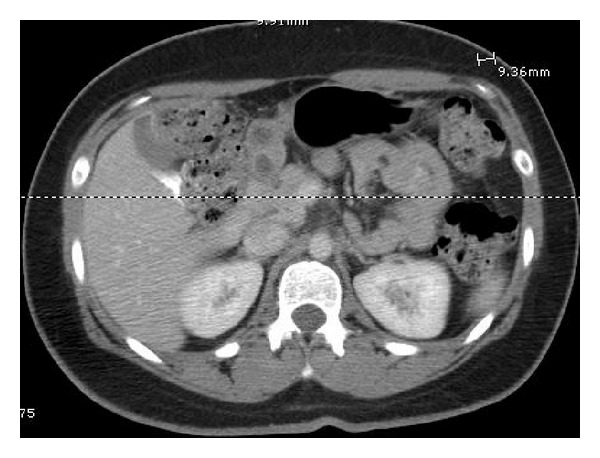
CT scan demonstrated four cystic lesions in the medial wall of distal antrum and pylorus measuring approximately one cm each, suggestive of duplication cysts.

 Diagnostic work up included abdominal CT scan, which demonstrated four cystic lesions in the medial wall of distal antrum and pylorus measuring approximately one cm each, suggestive of duplication cysts (Figure 4). 

 Upper GI endoscopy showed bulging of the gastric antrum and pylorus by an external compression without any mucosal abnormality. Endoscopic ultrasound showed multiple intramural cystic lesions measuring 3.5 × 2.5 cm in total dimension. The cysts appeared to be lined by mucosal layer with surrounding muscularis propria suggestive of duplication cysts. Fine needle aspiration was attempted but failed. 

 An exploratory laparotomy with antrectomy and truncal vagotomy with billroth II reconstruction were performed. 

 Patient's postoperative course was uneventful. She was discharged on postoperative day 10 and has been asymptomatic since then.

**Figure 5:**
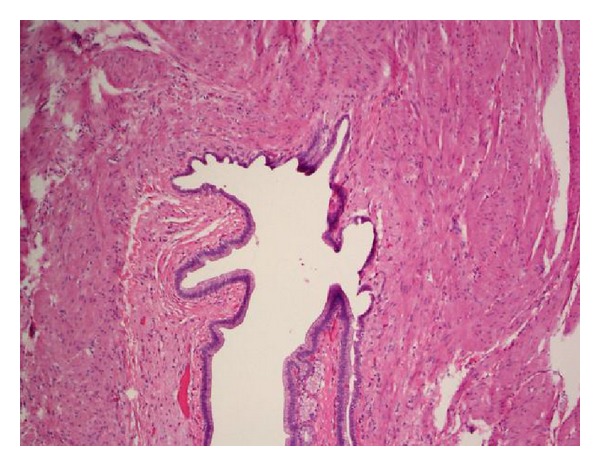
Photomicrograph: cyst wall was composed of mucosa, submucosa, and muscularis propria. Mucosa was predominantly of gastric type with small islands of pancreatic acini.

 Cut surface of specimen revealed two cysts filled with clear mucinous fluid measuring 2 cm and 1.3 cm in the greatest dimension. The inner surface of cysts was lined by pink-tan epithelium, and wall thickness was approximately 0.6 cm. There was no communication between the cysts and gastric segment. On microscopy, cyst wall was composed of mucosa, submucosa, and muscularis propria. Mucosa was predominantly of gastric type with small islands of pancreatic acini (Figure 5).

## 3. Discussion 

 Gastrointestinal duplication is a relatively rare anomaly that may occur at any level from oral cavity to rectum with ileum being the most common site. Duplication cysts of the stomach are quite rare, and most of them have been reported in children [1, 5, 6]. Duplication cysts of ileum are usually located on mesenteric border [7], whereas the usual location for gastric duplication cysts is along the greater curvature [4, 6, 7]. The duplication cyst is entirely separated from the adjacent bowel but shares a common wall [8]. 

 The essential criteria for diagnosis of a gastric duplication cyst are (a) the wall of the cyst is contiguous with the stomach wall; (b) the cyst is surrounded by smooth muscle, which is continuous with the muscle of the stomach; and (c) the cyst wall is lined by epithelium of gastric or any other type of gut mucosa [1, 4, 9].

 Our present cases fulfilled these criteria excluding other diagnoses.

 Gastric duplication cysts comprise 4% of all gastrointestinal duplications. Various other congenital anomalies such as alimentary tract duplications, esophageal diverticulum, or spinal cord abnormalities are encountered in up to 50% patients [8]. 

 These malformations are believed to be congenital, formed before the differentiation of epithelial lining, and therefore named for the organ with which they are associated [3, 10]. Duplications result from the disturbances in embryonic development, and various theories have been proposed for the actual mechanism. Bremer proposed the theory of errors of recanalization and fusion of longitudinal folds. He suggested that duplication cysts originated from the fusion of longitudinal folds allowing the passage of a bridge of submucosa and muscle at the second and third month of intrauterine life [5]. McLetchie suggested that adhesion of notochord and embryonic endoderm might not elongate as quickly as its surrounding structures, causing traction diverticulum leading to duplication cyst formation [5]. Other theories of enteric duplication include abortive twinning, persistent embryological diverticula, and hypoxic or traumatic events [5]. There is no single theory that is satisfactory for all types of duplications [5].

 Greater than 80% of gastric duplications are cystic and do not communicate with lumen of the stomach. The remainders are tubular with some communication [5]. The structure is defined as tubular when the lumen is contiguous and cystic when the lumen is not contiguous with stomach lumen [6]. The mucosal lining of duplication may be histologically similar to the segment of gut to which it is topographically related. However, some duplications may include lining from other segment of alimentary or respiratory tract. The presence of respiratory epithelium in the cysts of thorax, tongue, liver, and stomach suggests that the undifferentiated epithelium of foregut might undergo transition to differentiated specialized epithelium during embryonic period [5]. 

 Gastric duplications typically become symptomatic during childhood. 67% are diagnosed within the first year of life, and less than 25% are discovered after age 12 [4]. The duplication cysts of the stomach are usually diagnosed intra-operatively in adults [10]. In our first patient, the preoperative CT and MRI findings were interpreted as being most consistent with a pancreatic neoplasm, and diagnosis of GDC was suspected only during surgery.

 The clinical presentation of gastric duplication cysts can be highly variable and nonspecific ranging from vague abdominal pain to nausea, vomiting, epigastric fullness, weight loss, anemia, dysphagia, dyspepsia with abdominal tenderness and epigastric mass on physical examination [4, 10]. Because most cases occur along the greater curvature of the stomach, the cysts can potentially compress the adjacent organs such as pancreas, kidney, spleen, and adrenal gland. Accordingly, the differential diagnosis would include lesions arising from these organs [2]. The cysts may also be manifested by complications such as infection, gastrointestinal bleeding, perforation, ulceration, fistula formation, obstruction, compression, or carcinoma arising in the cysts [7, 8]. Up to 10% of gastric duplications may contain ectopic pancreatic tissue which may lead to pancreatitis and mimic a pancreatic pseudocyst [3, 8]. 

 Because of the rarity of adult gastric duplications, it is difficult to outline their natural history with certainty. As with the native gastric mucosa, the cyst lining may undergo erosions, ulceration, and regenerative changes. In noncommunicating cysts, increased fluid production may result in pressure-induced necrosis of the mucosa. These changes may lead to bleeding into the cyst or perforation into the peritoneal cavity. 

 Duplication cysts have the potential for neoplastic transformation. The production of oncofetal antigens raises the problem of a precancerous condition in long standing intestinal duplications [8]. Out of 11 reported cases of malignancy arising within the duplication cysts, 8 were adenocarcinomas [4]. Five of the carcinomas originated from gastric duplications. Adenomyoma arising from a gastric duplication has also been reported [4]. Malignancies arising from duplication cysts are likely to be present at advanced stages because of their unusual symptoms and difficulty of diagnosis [4]. 

 Although it is difficult to diagnose GDC preoperatively, recent imaging modalities have provided some informative findings. CT scan and endoscopic ultrasound (EUS) are the best ways to identify GDC [8]. Classically, radiographic studies show an intramural filling defect indenting the gastric contour [8]. Contrast-enhanced CT scan typically demonstrates GDC as a thick-walled cystic lesion with enhancement of the inner lining [2]. Calcification is occasionally observed on CT. These findings are of diagnostic significance for GDCs [2]. However, since mucinous cystic tumors of the pancreas also show similar radiological features, GDCs adjoining the pancreas are indistinguishable from pancreatic mucinous cystic tumors based on these CT findings. Moreover, because the wall is sometimes thin, enhancement of the inner cyst wall is not always demonstrated. Generally, MRI can provide additional information about the cyst content compared to CT scan. However, the nature of the fluid in the GDC was reported to differ in each case according to bleeding, chronic inflammation, or infection. Therefore, MRI seems to be of less significance than expected in diagnosing GDCs [2]. EUS is useful in distinguishing between the intramural and extramural lesions of the stomach. When EUS demonstrates a cyst with an echogenic internal mucosal layer and a hypoechoic intermediate muscular layer, the diagnosis of GDC is highly likely [2]. The role of EUS-guided FNA in GDC is uncertain because (a) the cytological features of GDC may closely resemble those of mucinous pancreatic neoplasms, and (b) GDCs with elevated levels of CEA and CA19-9 have been reported, mimicking mucinous pancreatic neoplasms [4, 8, 11]. 

 Complete removal is the treatment choice to avoid the risk of possible complications such as obstruction, torsion, perforation, hemorrhage, and malignancy [9, 10]. A noncommunicating GDC is classically treated by complete excision of the cyst and resection of the shared wall between stomach and the duplication cyst [8]. Communicating GDC usually requires no intervention when both gastric lumens are patent [8]. Drainage and marsupialization of the cyst have been suggested. However, marsupialization into the stomach exposes the unprotected mucosa of the cyst to gastric contents with the risk of ulceration [4]. Drainage procedures such as cystojejunostomy may be complicated by stenosis of the anastomosis or blind loop syndrome and therefore discouraged [4]. Furthermore, leaving the cyst in place is ill-advised given the potential for malignant transformation [4]. 

## 4. Conclusion

In summary, this unusual developmental anomaly should be included in the differential diagnosis of cystic masses of the gastrointestinal tract, and the possibility of malignancy should also be considered. While the diagnosis of gastrointestinal tract duplications may be suggested by imaging studies, more often the correct diagnosis is not established prior to surgery. Due to the risk of malignant transformation and other complications, GDCs should be treated surgically by complete resection.
